# Addressing the challenges of field notes in medical education: a qualitative study of resident experiences

**DOI:** 10.1186/s12909-025-07578-w

**Published:** 2025-07-01

**Authors:** Ryan S. Huang, Tushar Sood, Matthew Nelms, Lauren Wintraub, Fok-Han Leung

**Affiliations:** 1 Temerty Faculty of Medicine, University of Toronto, Toronto, ON Canada; 2 Department of Family and Community Medicine, University of Toronto, Toronto, ON Canada; 3 Health Centre at 80 Bond, St. Michael’s Hospital, 80 Bond Street, Toronto, ON M5B1X2 Canada

**Keywords:** Field notes, Feedback, Competency-based medical education

## Abstract

**Background:**

Evaluating resident physicians’ competencies is critical in medical education to ensure high standards of patient care and professional development. Field notes are increasingly used as reflective tools in postgraduate medical education. Despite their growing use, skepticism about their effectiveness persists. This study aims to identify challenges with learner engagement in field notes and gather suggestions for operational improvements.

**Methods:**

A qualitative study was conducted in the Department of Family & Community Medicine at the University of Toronto. Semi-structured interviews were conducted with seven postgraduate year one and year two family medicine residents. The interviews focused on residents’ experiences and challenges with field notes. Data were analyzed using inductive thematic analysis to develop a comprehensive codebook, in alignment with Braun and Clarke’s framework.

**Results:**

Several key challenges with the use of field notes were identified including the redundancy of feedback, sporadic utilization, and time constraints for preceptors. Residents also expressed uncertainty about the expectations for using the tool and identified it as complex and cumbersome. Operational suggestions for improvement included the development of a mobile-friendly platform, streamlined functionality, a standardized and integrated feedback system, and clearer guidelines for use.

**Conclusions:**

The study highlighted significant challenges in the use of field notes within family medicine training programs and underscored the need for technological and procedural innovations to improve their effectiveness. Addressing these challenges through user-friendly design, clear guidelines, and integrated support systems could transform field notes into a more robust tool for competency-based medical education, benefiting residents, preceptors, and the broader medical community.

**Supplementary Information:**

The online version contains supplementary material available at 10.1186/s12909-025-07578-w.

## Introduction

In medical education, evaluating resident physicians’ competencies is crucial for maintaining high standards of patient care and advancing professional skills. Competency-based assessments, particularly field notes, are increasingly utilized in postgraduate medical education across Canadian family medicine programs [1]. 

Field notes are structured documentation tools used to capture observations of clinical encounters and provide timely, specific feedback to learners [2]. In residency training, they serve as both assessment and teaching instruments. Unlike traditional summative evaluations that occur at the end of rotations, field notes are designed to be collected throughout training to document progress across various domains of competency [3]. These brief narrative assessments typically include documentation of the clinical scenario, observed strengths, suggestions for improvement, and often include a rating scale to indicate level of supervision required [4]. The intent is to create a longitudinal record of resident performance that demonstrates growth over time and highlights areas needing focused attention. Field notes can be initiated by either the resident or preceptor, may be completed on paper or electronically, and ideally are discussed face-to-face before being formalized in writing. They represent a cornerstone of workplace-based assessment within competency-based medical education frameworks, particularly in Canadian family medicine programs where they have been widely adopted [3]. 

Serving as a reflective tool, field notes enable both residents and preceptors to evaluate clinical performance. This process aids residents in making actionable improvements based on thorough feedback [5, 6]. However, despite their growing use, significant skepticism remains about their effectiveness. A study by Matthew et al. revealed that nearly half of the surveyed residents doubted the effectiveness of field notes [7]. This study aims to identify the challenges with learner engagement and gather operational suggestions to improve the tool. The findings will contribute to academic discussions and guide the continual implementation of field notes in postgraduate medical education programs.

## Methods

### Study design and setting

This qualitative study was conducted within the Department of Family & Community Medicine (DFCM) at the University of Toronto. By employing a qualitative design, we aimed to capture in-depth insights into the residents’ experiences with the field notes tool.

### Data collection

Participants were recruited from the cohort of postgraduate year one (PGY-1) and two (PGY-2) family medicine residents currently enrolled in the program at the University of Toronto. Recruitment was conducted through email to ensure a diverse representation in terms of residency year, gender, and prior experience with the field notes tool. Participation was voluntary and recruitment continued until thematic saturation was reached. We conducted semi-structured interviews between December 2022 and August 2023, using a flexible yet targeted approach to explore residents’ views while adapting to the natural flow of conversation. The interview guide, developed to probe into residents’ experiences with field notes and avenues for potential enhancements, served as a foundational script (Supplementary Material 1). In accordance with contemporary research adaptations necessitated by the COVID-19 pandemic, interviews were conducted via Zoom. This method ensured the safety of all participants while maintaining the integrity of the data collection process. Interviews were audio-recorded with the consent of the participants and then de-identified to maintain confidentiality.

### Data analysis

Transcripts from the interviews were analyzed using inductive thematic analysis to identify patterns and themes in the data. Two medical students at the University of Toronto (R.S.H., T.S.) independently reviewed the verbatim transcripts to pinpoint key words, phrases, and latent content. These preliminary findings were then discussed in a group setting to achieve consensus on a coding schema. Discrepancies in interpretations were addressed through constructive dialogue and, when necessary, arbitration by a third author (F.L.), who is a faculty member within the DFCM with experience supervising residents and completing field notes. A codebook was crafted to facilitate the systematic identification, organization, and reconciliation of themes both independently and as a collective. Through iterative group discussions, patterns were identified, categorized and developed into themes. To substantiate the identified themes, direct quotes from the transcripts were extracted. This process was conducted in alignment with Braun and Clarke’s framework for inductive thematic analysis (familiarization with the data, generating initial codes, searching for themes, reviewing themes, defining and naming themes, and producing the report) [8]. 

### Ethical considerations

Ethical approval was obtained from the University of Toronto Research Ethics Board. Informed consent was acquired from all participants. This study was conducted in compliance with the Declaration of Helsinki.

## Results

### Participant characteristics

Seven family medicine residents participated in semi-structured interviews (Table 1).

**Table 1:** Demographics of included participants

Interviewee	Gender	Medical School	Postgraduate Year	Site of Training
1	M	University of Toronto	PGY-1	Scarborough
2	F	University of Calgary	PGY-2	St. Michael’s
3	F	University of British Columbia	PGY-2	Markham
4	F	University of Toronto	PGY-2	Mt. Sinai
5	F	University of Manitoba	PGY-1	Mt. Sinai
6	M	McMaster University	PGY-2	North York
7	M	University of Toronto	PGY-1	Trillium

### Theme 1: residents acknowledge benefits of field notes

Residents identified several positive aspects of the field notes tool that enhanced their educational experience (Table 2). The accessibility of field notes was particularly valued, with residents appreciating how easily they could review their feedback throughout their training. One resident noted, “I liked how you have very easy access to all the field notes throughout residency. It wasn’t just like the latest one. I could very easily review any of the ones I wanted to” (P4). Participants also recognized the potential of field notes for providing regular feedback across diverse clinical scenarios, with one stating, “I like how you get regular feedback on a variety of cases using the tool” (P1).

**Table 2:** Illustrative quotes regarding residents’ perceived benefits and drawbacks of the field notes tool

Residents acknowledge benefits of field notes	
Field notes are easily accessible	“I thought it was easy to access and understand what was going on.” (P2)
	“I liked how you have very easy access to all the field notes throughout residency. It wasn’t just like the latest one. I could very easily review any of the ones I wanted to.” (P4)
Field notes have potential	“I like how you get regular feedback on a variety of cases using the tool.” (P1)
	“I think it’s a great tool. Generally, I think field notes are great when used properly.” (P6)
Residents enjoy the inclusion of Likert scales	“I think the rating scale is pretty standard and that’s good because you of see where someone is in terms of how much supervision is required.” P3)
	“I think the Likert scales make it really simple that way.” (P7)
Residents believe field notes contribute to their professional growth	“…helps with direction in terms of career… that was helpful in terms of deciding fellowship.” (P2)
	“It’ll help you grow as a resident.” (P7)
Residents believe field notes contribute to their learning	“You will know what you are doing well, and you will still know what you could improve on. So, I thought that was helpful.” (P6)
	“So even if it was a bad field note, like it literally doesn’t matter at the end of the day. So, there was never any embarrassment. There was never any like, oh no, like what does this mean for residency. It was always like taken as like this is a learning opportunity.” (P5)
**Residents are critical of field notes, highlighting some limitations**	
Residents think the tool can be extra work and not adding enough	“Oftentimes, it gets pushed back and forgotten about, more so feels like a responsibility on the resident to go and ask for them, which can feel annoying sometimes” (P4)
	“I can see the value of it, but I think it can be artificial sometimes because then some residents are like scrambling last minute to try to ask any preceptor to observe them.” (P1)
	“The process is just cumbersome in general. It’s like an extra thing people have to do and on top of patient care.” (P5)
	“I don’t know if necessarily having them in the form of field notes actually contributed to the fact that that feedback helped me out.” (P7)
Residents believe preceptors do not have enough time to complete the field notes	“Broadly speaking, time is a barrier to complete the field notes.” (P1)
	“It’s just like having the time to do it.” (P2)
Residents are unsure of what is expected of them using the tool	“I think there was a little bit of stress associated with the tool, mainly because there was no concrete idea of how many field notes you are supposed to have” (P6)
Residents feel that they are insufficiently prepared	“Just overall, it was a lot less detailed of an introduction and made it a little harder to use, especially at the beginning.” (P5)
	“I felt like in terms of how to initiate a field note, it was kind of reviewed in the beginning of residency, but as you go forward you kind of forget how to do that kind of stuff.” (P3)
Residents feel that their preceptors are insufficiently prepared	“Sometimes you’ll send the field notes and it’ll sit in their inbox for months and months and no one really bothers to look at it.” (P1)
	“I think a lot of preceptors didn’t know about it.” (P4)
Residents believe there are too many feedback platforms used	“There’s too many platforms for me to have to use to get feedback” (P2)
	“Why is there no like integration button, why is everything on different platforms?” (P6)
Feedback received through field notes may be inadequate	“I thought that sometimes when the field notes were done there wasn’t either enough info or it wasn’t specific enough. Like for example, if they said excellent communication, it’s sometimes helpful to know like, well what made it excellent?” (P5)
Different physicians may place different values on field notes	“I found like different staff took them more seriously as compared to others” (P6)
	“A lot of residents were not engaged in the field notes. It was just like another thing that they had to do” (P7)
Residents believe field notes are not suited for feedback on an ongoing, encounter-by-encounter basis	“So, it’s the time issue, I know the field note tool is supposed to be a remedy to that because it’s online, but again, there’s even that issue where sometimes it doesn’t even get done online.” (P3)
	“Obviously clinics are busy, so it’s hard to even remember at the end of clinic to do a field note” (P4)
Some learners find the field notes tool to be difficult to use	“The navigation of the site is kind of difficult… I get to go through multiple pages to find a field note or maybe I’m not well versed on the software.” (P1)

*Note: Grey bar headings represent major themes identified in our analysis. The left column presents specific codes within each subtheme*,* with corresponding illustrative quotes in the right column.*

The inclusion of standardized rating scales was another feature residents found beneficial. These scales helped them understand their performance level and supervision requirements in concrete terms. As one resident explained, “I think the rating scale is pretty standard and that’s good because you see where someone is in terms of how much supervision is required” (P3). Beyond these structural benefits, residents believed field notes contributed meaningfully to their professional development. One participant shared how field notes “helps with direction in terms of career… that was helpful in terms of deciding fellowship” (P2). Residents also valued the learning opportunities provided by field notes, appreciating how the tool created a structured approach to receiving constructive feedback. The formative nature of field notes reduced anxiety about performance, as one resident explained: “So even if it was a bad field note, like it literally doesn’t matter at the end of the day. So, there was never any embarrassment… It was always like taken as like this is a learning opportunity” (P5).

### Theme 2: residents are critical of field notes, highlighting some limitations

Despite recognizing potential benefits, residents described several challenges that diminished the field note tool’s utility. Many considered it an additional administrative burden amidst their already demanding schedules. One resident remarked, “The process is just cumbersome in general. It’s like an extra thing people have to do and on top of patient care” (P5). This sense of burden was exacerbated when field notes seemed redundant with verbal feedback already received during clinical encounters. As one resident noted, “I don’t know if necessarily having them in the form of field notes actually contributed to the fact that that feedback helped me out” (P7). The sporadic use of field notes further diminished their effectiveness. Rather than serving as a consistent, ongoing feedback mechanism, residents reported that field notes were often completed in batches toward the end of rotation blocks. Time constraints emerged as a significant barrier, particularly for preceptors. As one resident observed, “Broadly speaking, time is a barrier to complete the field notes” (P1). These constraints often led to rushed feedback that lacked depth and specificity.

Uncertainty regarding expectations created additional stress for residents. One participant shared, “I think there was a little bit of stress associated with the tool, mainly because there was no concrete idea of how many field notes you are supposed to have” (P6). Technical and usability issues further complicated the process. Residents described a cumbersome experience of chasing preceptors for completion and navigating multiple software platforms. One resident explained, “The navigation of the site is kind of difficult… I get to go through multiple pages to find a field note or maybe I’m not well versed on the software” (P1). Inconsistent engagement among faculty also affected the tool’s perceived value, with one resident noting, “I found like different staff took them more seriously as compared to others” (P6).

### Theme 3: Residents would like an improved field notes platform

Residents provided thoughtful suggestions for enhancing the technological aspects of the field notes system (Table 3). A recurring recommendation was the development of a mobile-friendly platform that would streamline the process. One resident emphasized, “It would be easier if the system was designed like an app where you just pull it up and fill it out, boom, boom and it’s done” (P6). Another echoed this desire for simplified access, stating, “I would love it if it was something that’s done through an app that doesn’t require as many steps to complete” (P5).

**Table 3:** Illustrative quotes regarding resident suggestions to improve the field notes tool

Residents would like an improved platform	
Residents prefer a mobile-friendly platform	“It would be easier if the system was designed like an app where you just pull it up and fill it out, boom, boom and it’s done” (P6)
	“I would love it if it was something that’s done through an app that doesn’t require as many steps to complete” (P5)
	“I wonder like if it’s possible that it could be turned into an easier app.” (P7)
Residents seek streamlined, effective functionality	“Streamline the process a little bit to, so you can have it done quickly and on the go” (P2)
	“As long as we can ease the process, you have to make it easy for preceptors to give feedback” (P3)
Residents prefer a standardized and integrated platform	“I just wish it was all in one area” (P4)
	“It would be great if it was all the feedback was centralized, so then we can get like a summary of all the different types of feedback, in one page” (P6)
	“Maybe with some fine tuning for the standardization, it’ll be even better” (P2)
**Improvements to processes and communication may enhance their experience**	
Clarity is needed on how they should use the tool	“Better if the objectives were clearly defined in terms of what you’re expecting… make them a bit more specific” (P4)
	“Clearly you don’t need like a certain number of field notes to graduate because I didn’t have that many and I passed. I think the DFCM just should set the expectations clearly on what exactly they need, how many need to be done.” (P3)
	“I think requirements are helpful because I think that it’s not fair for some residents to have their feedback tracked and others not.” (P5)
Learners have diverse opinions on how field notes should be initiated	“More of like preceptor driven feedback versus the resident having to ask for it.” (P1)
	“When preceptors are actually evaluating an encounter, making sure that they’re evaluating it from start to finish, not just snippets.” (P7)
	“The best thing would be to put more responsibility on preceptors to actually remember to do these regularly, and also are like, not only done for exceptionally good encounters.” (P3)
Learners believe adjacent supports will help improve field notes	“One of the things I would change with the field note is that it should be discussed before they’re submitted.” (P3)
	“Maybe if there was like a document about how to navigate the website might be helpful.” (P2)
	“Unless there’s like some sort of protected time in clinic before preceptors go home, I don’t really see this changing unless somehow the process is simplified.” (P4)
	“If there’s incentives, like what motivates preceptors to complete these notes.” (P6)

*Note: Grey bar headings represent major themes identified in our analysis. The left column presents specific codes within each subtheme*,* with corresponding illustrative quotes in the right column.*

Participants also advocated for more streamlined and efficient functionality to reduce the time burden. One resident suggested the need to “streamline the process a little bit too, so you can have it done quickly” (P2). Recognizing the importance of preceptor engagement, another noted, “As long as we can ease the process, you have to make it easy for preceptors to give feedback” (P3). Integration and standardization were also prioritized in residents’ suggestions. Many expressed frustration with having to navigate multiple platforms for different types of feedback and recommended centralizing these systems. As one resident explained, “It would be great if it was all the feedback was centralized, so then we can get like a summary of all the different types of feedback, in one page” (P6).

### Theme 4: Improvements to processes and communication may enhance their experience

Beyond technological enhancements, residents identified process and communication improvements that could transform their experience with field notes. Clarity of expectations emerged as a critical need. One resident highlighted, “Better if the objectives were clearly defined in terms of what you’re expecting… make them a bit more specific” (P4). Another emphasized the need for explicit guidelines, stating, “Clearly you don’t need like a certain number of field notes to graduate because I didn’t have that many and I passed. I think the DFCM just should set the expectations clearly on what exactly they need, how many need to be done” (P3). Participants offered diverse perspectives on field note initiation. Some preferred shifting responsibility toward preceptors, with one resident suggesting, “More of like preceptor-driven feedback versus the resident having to ask for it” (P1). Others focused on improving the quality and comprehensiveness of evaluations, recommending that “when preceptors are actually evaluating an encounter, making sure that they’re evaluating it from start to finish, not just snippets” (P7).

Additional support systems were suggested to enhance the field notes process. Residents valued discussion-based approaches, with one proposing, “One of the things I would change with the field note is that it should be discussed before they’re submitted” (P3). Practical aids such as navigation guides were recommended, along with structural changes like protected time for preceptors. As one resident realistically observed, “Unless there’s some sort of protected time in clinic before preceptors go home, I don’t really see this changing unless somehow the process is simplified” (P4).

## Discussion

Residents identified key operational challenges that compromise the field note tool’s effectiveness. These findings contribute to the ongoing discussion on assessment tools in medical education, emphasizing the need to align assessment methods with educational goals while ensuring practicality and ease of use.

At the University of Toronto’s DFCM residency program, field notes are implemented through a web-based platform that residents and preceptors access through the program’s learning management system. Residents are expected to collect approximately 2–3 field notes per week across different clinical domains. The process typically begins with the resident initiating a request for observation, after which the preceptor observes all or part of a clinical encounter. Feedback is ideally provided verbally immediately following the encounter, and then documented electronically within the platform. The field note form includes sections for describing the clinical scenario, documenting specific feedback on strengths and areas for improvement, and rating the resident’s performance using a 5-point entrustment scale ranging from ‘close supervision required’ to ‘able to supervise others.’ Residents receive an email notification when preceptors complete a field note, allowing them to review the feedback and reflect on their performance. Program directors periodically review residents’ collected field notes to identify patterns and inform summative assessments. While there is no specific minimum number of field notes required for promotion, residents are encouraged to gather sufficient documentation to demonstrate progress across all competency domains.

While the benefits of field notes—such as promoting reflective practice and guiding residents through a competency-focused curriculum—are well-acknowledged [9–11], our findings indicate that the timing and consistency of feedback delivery often fail to meet the immediacy required by competency-based medical education. Feedback was frequently postponed until the end of clinical rotations, limiting its impact on immediate learning and adaptation. Furthermore, criticisms regarding the redundancy of field notes, their sporadic use, and real and perceived administrative burdens highlight the discrepancy between the goals of competency-based assessments and their practical execution, especially given medical residents’ demanding schedules [12]. The perception of field notes being burdensome is exacerbated by preceptors’ time constraints, which compromise feedback’s timeliness and specificity, thereby reducing the educational value of the tool [13–15]. These findings underscore the challenge of translating the theoretical advantages of comprehensive assessment tools into practice.

The identification of these challenges points towards a critical need for innovation in the design and deployment of assessment tools within medical education. A streamlined, user-friendly system that minimizes administrative burdens while maximizing the opportunities for regular, meaningful feedback could significantly enhance the utility and acceptance of field notes among residents and preceptors alike. Such a system would ideally integrate seamlessly into the daily workflows of medical education, ensuring that the process of documenting and reflecting on clinical experiences does not detract from the core educational objectives of learning and improvement. Moreover, the call for a more user-friendly and efficient system for field notes reflects a broader recognition of the importance of technology and design thinking in medical education [16–18]. By adopting principles of usability, accessibility, and integration, educational tools can be developed to support the complex process of learning and assessment in clinical settings more effectively [19–23]. This approach not only addresses the practical challenges identified by residents and preceptors but also aligns with the evolving landscape of medical education, which increasingly recognizes the value of adaptive, learner-centered strategies that support the continuous development of clinical competencies.

The limitations inherent in our study warrant consideration. Primarily, the relatively small sample size, confined to residents within a single department at one institution, poses a significant constraint on the generalizability of the results. Therefore, the insights garnered from our cohort may not fully encapsulate the diverse range of experiences and perceptions that exist among all medical residents. The thematic coding was conducted primarily by two medical students at the institution, which, while offering valuable insights from learners’ perspectives, may have introduced certain biases in data interpretation. To enhance reflexivity, the team engaged in regular discussions to critically examine how their perspectives, prior experiences, and assumptions could shape data interpretation. We maintained reflexive memos documenting emerging interpretations and potential biases, challenging each other’s readings of the data and seeking alternative explanations before finalizing themes. Members with field note experience provided contextual insights during team discussions while being mindful not to impose their preconceptions. Through this iterative process of questioning our assumptions and returning to the data, we worked to ensure our themes authentically represented participants’ experiences rather than our own expectations. Additionally, the qualitative nature of our study, while invaluable for obtaining in-depth, nuanced understandings of residents’ experiences and perceptions, also means that the perspectives captured may not be representative of all residents’ experiences. Qualitative research is inherently subjective and seeks to explore complex phenomena within specific contexts rather than to generalize findings across populations. As such, the rich, detailed insights provided by the participants in our study offer a deep dive into the specific challenges and opportunities for enhancing feedback practices within their context but may not encompass the full spectrum of opinions, experiences, and practices that exist within the wider medical education community. Furthermore, our study did not include member checking, which should be considered in future studies.

## Conclusion

Our study suggests that there may be technical and policy-driven challenges that affect the utility of field notes in competency-based medical education. Participants identified potential areas for improvement, including enhanced technological platforms and clearer operational guidelines. These insights, while drawn from a limited sample, point to possible directions for optimizing field notes in medical education. Future research with larger, more diverse samples across multiple institutions would be valuable to determine the generalizability of these findings and develop evidence-based strategies for enhancing field note systems.

## Electronic supplementary material

Below is the link to the electronic supplementary material.

Supplementary Material 1

## Data Availability

The datasets used and/or analysed during the current study are available from the corresponding author on reasonable request.

## Declarations

### Clinical trial number

N/A. This study is not a clinical trial.

## Abbreviations

DFCM: Department of Family & Community Medicine
PGY-1: Postgraduate Year One
PGY-2: Postgraduate Year Two
COVID-19: Coronavirus Disease 2019
