# Supplementary material for: Addressing the challenges of field notes in medical education: a qualitative study of resident experiences
Source: BMC Med Educ. 2025 Jul 1;25:883. doi: 10.1186/s12909-025-07578-w (PMC12211422; doi:10.1186/s12909-025-07578-w)
Supplement: Supplementary file 1 — Supplementary Material 1 [file 12909_2025_7578_MOESM1_ESM.docx]

**Family Medicine Residents’ Perspectives on Field Notes**

**Key Informant Interview Guide**

**Part A**

*Greetings*

“Hello! My name is {...} and I am a researcher with Dr. Leung from the University of Toronto medical school. We are interested in interviewing current Family Medicine residents to gauge your understanding of the field note assessment tool and better understand your experiences with using it. We hope that the information that we gather will help improve this tool for future residents.”

*Explanations*

“Thank you for meeting with me today. I am interested in learning about your general experience in the residency program thus far, your thoughts on feedback and how it is given, and the field note assessment tool and its utility. The questions I am going to ask don’t have right or wrong answers. Remember that this conversation is completely confidential. You can choose to terminate this interview at any point, and we can skip any question you prefer not to answer. I will also be taking some notes as we speak and recording this interview for transcription later. Are you okay to continue?”

{If yes, start recording now} “I am going to start recording now.”

**Part B.**

**Opening Questions**

- What is your name?
- Can you give me a brief overview of your journey to the U of T Family Medicine residency program?
  - Where did you complete your medical school training?
- What year of residency are you in?
- What site are you mainly located at?
- What is your current rotation?

**Key Question:** What are the perspectives of Family Medicine residents regarding the field note assessment tool used in the post-graduate medical education program?

1. **General thoughts about feedback**

- **What do you think is the value of feedback in medical training, specifically residency programs?**
  - Probe: How do you think it contributes to your program?
- **Can you tell us about the ways in which you have received feedback throughout the course of your residency so far?**
  - Probe: Who provides this feedback (preceptors, peers/colleagues, allied health, self)? Do you weigh feedback differently based on who it’s coming from?
  - Probe: What format is this feedback provided in (verbal, written, informal, evaluation forms)?
  - Probe: How often are there opportunities to give/receive feedback (daily, weekly, monthly)? Do you think that this is enough? Why or why not?
  - Probe: Which clinical settings is feedback primarily given in (patient communication, resident-preceptor interaction, clinical skills)?

1. **Resident Field Notes**

- **Can you describe your understanding of the Field Note Assessment Tool?**
  - Probe: How often is it utilized for your feedback? What prompts you to use it this often?
  - Probe: What are some benefits to using this tool?
  - Probe: What are some limitations of this tool?
- **Think of your most recent experiences using the Field Note Assessment Tool. How did they go?**
  - Probe: Who do you usually ask to complete the Field Note? Why?
  - Probe: How would you describe your relationship with the person or faculty who gave you feedback?
  - Probe: How difficult was it to get them to complete the assessment? What do you think made it difficult?
  - Probe: How did you feel receiving this feedback? Why?
  - Probe: How do you think the person or faculty felt when giving feedback? Why?
- **If you completed the self-assessment section, what did you notice about yourself as you completed it? (This is the most important section, make sure to probe and expand on answers here)**
  - Probe: What motivates you to complete the self-assessment (fulfilling a quota set by the program, learning purposes)?
  - Probe: How did your self-assessment differ from the faculty’s feedback? How did you feel about this?
    - Did it differ in terms of areas that were assessed (patient interactions vs resident-preceptor interactions, CANMEDS roles)? Did it differ in terms of the quality of responses?
  - Probe: Would you feel differently if the Field Note didn’t contain the self-assessment component? If so, how?
- **How does this feedback contribute to your training/learning?**
  - Probe: How does it impact your performance during future clinical encounters?
  - Probe: How does it contribute to your reflections on your learning and practice?
  - Probe: How meaningful is the feedback given to you? In what ways (actionable takeaways, constructive criticism, support/praise for current actions)?

1. **Way forward**

- **How do you think the Resident Field Note assessment tool can be improved for the future?**
  - Probe: What would you like changed about the tool?
  - Probe: What about this tool should remain the way it is?
- **How do you think Field Note completion rates by residents, faculty or others can be increased?**
  - Probe: What are some barriers to completion for each of these parties?
- **In your opinion, what are the best ways to receive feedback that will contribute most effectively to your learning?**

**Part C.**

**Summary**

- Is there anything else that you would like to say about the topics we have discussed today?
- Is there anything else you feel is important for us to know about your experience?

*Closing*

“Great! That is the end of our interview. I would like to thank you for your time and for sharing your experiences with us. If you have any further questions or comments, please do not hesitate to reach out.”
